# Integrating Care for Medical and Mental Illnesses

**Published:** 2006-03-15

**Authors:** Katharine H McVeigh, Lloyd I Sederer, Lynn Silver, Joslyn Levy

**Affiliations:** Bureau of Epidemiology Services, New York City Department of Health and Mental Hygiene; New York City Department of Health and Mental Hygiene, New York, NY; New York City Department of Health and Mental Hygiene, New York, NY; New York City Department of Health and Mental Hygiene, New York, NY

Since 2002, the New York City Department of Health and Mental Hygiene (DOHMH) has conducted an annual Community Health Survey (CHS) through which a random-digit–dialed sample of approximately 10,000 adults in 34 New York City neighborhoods are interviewed by telephone. We ask respondents about their health, mental health, behavioral risks (e.g., smoking, diet, exercise), and functioning. Each year the information we gather demonstrates, to paraphrase former Surgeon General David Satcher, that there is no health without mental health.

From the New York City CHS, we have established that New Yorkers who report nonspecific psychological distress (NPD) — as measured using Kessler's K6 scale (1) — have more problems with their physical health than New Yorkers who do not report NPD. In addition, New Yorkers with NPD are more likely to be smokers, to be sedentary, and to have a poor diet. They are also far more likely to have hypertension, hyperlipidemia, obesity, asthma, and diabetes (2). In this editorial, we summarize the evidence for the link between medical and mental disorders and discuss what DOHMH is doing to improve the health of New Yorkers with depressive illness.

Two review articles (3,4) present overwhelming evidence that mental disorders and medical illnesses are strongly linked. Medical illnesses such as cardiovascular disease, diabetes, asthma, and cancer are associated with mental illnesses, and the more serious the medical condition, the more likely it is that the patient will experience a mental illness. Individuals with depressive disorders are about twice as likely to develop coronary artery disease, twice as likely to have a stroke, more than four times as likely to have a myocardial infarction (MI), and four times as likely to die within 6 months of an MI as people without depressive disorders. Depression is a common poststroke condition, and effective treatment of depression can improve cognitive functioning and survival. People with diabetes are two times as likely to have depression as the general population, and the presence of depression as a comorbidity to diabetes is associated with poor adherence to medication regimens, greater complications of diabetes, increased numbers of emergency room visits, and poorer physical and mental functioning. Health care expenditures are more than four times greater for people with diabetes who have depression than for people with diabetes who do not have depression. Symptoms of depression and anxiety are common (up to 50%) in people with asthma. The presence of depressive symptoms increases as the frequency of attacks increases. People with asthma who have mental disorders are far more likely to visit primary care physicians and emergency departments and to be hospitalized than people with asthma who do not have mental disorders. Individuals with cancer commonly have mental disorders, especially depression; effective treatment of depression is associated with decreases in pain and symptoms as well as improved immune functioning. Interestingly, improved hemoglobin levels in people with cancer have been reported to improve depressive symptoms, reminding us again about the link between medical and mental disorders. As many as one third of individuals with HIV/AIDS have clinically significant depressive symptoms or a diagnosable mood disorder. People who are HIV-positive have twice the risk of developing major depression as people who are HIV-negative. Notably, HIV-positive women are more likely to have depression than HIV-positive men. Bipolar disorder (manic depressive disease) is also found commonly in people with HIV/AIDS and is associated with poor adherence to antiretroviral treatment and with risky sexual and drug-abusing behaviors. We also know that depression and poor social support are associated with a more rapid progression from HIV infection to AIDS.

Evidence is also accumulating rapidly that novel antipsychotic medications are associated with complications such as obesity, high blood glucose levels, and diabetes. The link between medical and mental illnesses thus extends to treatments, not just the diseases. Chronic pain, Alzheimer's disease and Parkinson's disease, epilepsy, and obesity are other conditions in which mental and medical disorders routinely coexist and in which the presence of a mental disorder impairs functioning and effective disease management (4).

Finally, people with mental disorders also have impairments in everyday functioning and have greater difficulty following medical regimens, leading to poorer response (3,4). Moreover, people with mental disorders are more likely to smoke and abuse alcohol and drugs, leading to greater risk for medical illness and poor adherence to medical treatments (2).

Despite this compelling evidence, primary care practitioners in the United States have yet to successfully detect mental disorders and provide beneficial treatments for most of their patients with mental disorders, especially those with depression. The National Comorbidity Survey Replication, conducted between February 2001 and April 2003, demonstrated that 60% of individuals with a mental disorder received no treatment in the 12 months before the survey, and of individuals who did receive care, only one third met criteria for minimally adequate care (5). In other words, four out of five people in the United States with treatable and often debilitating mental illness do not receive effective treatment. In addition, substantial lags exist between the onset of mental disorders and their diagnosis and treatment, especially among the poor, the poorly educated, and people of color.

For years, efforts to improve detection and treatment of mental illness by primary care providers through education and exhortation have not succeeded. Something is evidently wrong with our approach, because primary care physicians know and care about the problem of mental illness. As with other chronic illnesses, a new approach focused on systematic changes in the delivery of care was needed. The Chronic Care Model (6) identifies critical areas of intervention to improve care for illnesses, including depression. Among these are attention to the design of the delivery system, clinical information and decision support, and self-management for patients. We have used this model in our planning.

In 2004, as a part of Take Care New York (7), a citywide initiative to improve the health of New Yorkers, DOHMH began to work with local primary care providers to integrate depression screening and management into primary care delivery systems, including the use of the electronic health record. Medical practice relies on quantifiable measures of a disease; patients are given a set of numbers to represent their weight, pulse, blood pressure, hemoglobin and hematocrit, electrolytes, and lipid levels. Diabetic management now has adopted the hemoglobin A1c as a standard metric for control. Physicians manage patient care using a number or target, and patients can relate a number to the severity of their condition. For example, if your blood pressure is 190/120, you know that you are in trouble, and your physician is driven to get those numbers back into the normal range. But to date, a quantifiable measure for a mental disorder has not been introduced systematically into primary care practice at a large population level (although fine examples exist in a few innovative health maintenance organizations and other settings).

We believe that quantifiably measuring and monitoring depression in primary care settings can improve its detection and management. We seek to change the landscape of practice and to enable patients and practitioners to close the gap between mental and medical disorders. We have selected the Patient Health Questionnaire-9 (PHQ-9) (8), a depression self-report scale, as our recommended tool for primary care practitioners. This 9-item questionnaire is specific to depression, sensitive, and available in many languages. The PHQ-9 yields a score from 0 to 27, with a score of 10 or higher likely to indicate a depressive disorder. The PHQ-9 should be readministered 12 weeks or more after the beginning of treatment for depression. When care is effective, there is a reduction in the patient's PHQ-9 score, analogous to a reduction in blood pressure, that both physician and patient can follow. Our goal is to normalize the detection and treatment of depression and in so doing make a difference in the health of our community.

Our depression initiative has begun in New York City municipal and voluntary hospitals and community health centers, and we are planning its introduction in university settings. In addition, this year, DOHMH will be visiting all primary care physicians in our highest-risk communities with "public health detailing" (9) to make recommendations and instruct them on depression screening and to supply clinical management tools such as guidelines and patient self-care techniques. We will also be instituting a public relations campaign with the theme, "Have you asked your doctor about a test for depression?"

Detection and management of depression in primary care practice can pose significant challenges to practicing physicians with too little time and often limited training in managing this prevalent condition. But we would not be deterred were the condition an infectious, a malignant, or an endocrine disease that was profoundly underdetected and undertreated. We should not be deterred by the challenge of detecting and treating depression, either, with its recognized morbidity and mortality. Primary care physicians have learned to work with diabetes and heart disease and only refer complex or treatment-resistant cases to specialty care. The same principles already familiar to primary care physicians can be applied to mental health conditions such as depression and include screening, a quantitative measure of the disease state, treatment (including medication) guidelines, brief counseling, and patient self-management.

The World Health Organization has defined health as a "state of complete physical, mental and social well-being and not merely the absence of disease or infirmity" (10). Physical and mental diseases are inextricably bound, and improving one improves the other. Improved mental and physical health will improve quality of life and social well-being. The evidence tells us what we need to know; the work ahead is to turn evidence into everyday practice.

## References

[1] Kessler RC, Barker PR, Colpe LJ, Epstein JF, Gfroerer JC, Hiripi E (2003). Screening for serious mental illness in the general population. Arch Gen Psychiatry.

[2] McVeigh KH, Mostashari F, Wunsch-Hitzig RA, Kuppin SA, King CG, Plapinger JD (2003). There is no health without mental health. NYC Vital Signs.

[3] Chapman DP, Perry GS, Strine TW (2005). The vital link between chronic disease and depressive disorders. Prev Chronic Dis.

[4] Evans DL, Charney DS, Lewis L, Golden RN, Gorman JM, Krishnan KR (2005). Mood disorders in the medically ill: scientific review and recommendations. Biol Psychiatry.

[5] Wang PS, Berglund P, Olfson M, Pincus HA, Wells KB, Kessler RC (2005). Failure and delay in initial treatment contact after first onset of mental disorders in the National Comorbidity Survey Replication. Arch Gen Psychiatry.

[6] Bodenheimer T, Wagner EH, Grumbach K (2002). Improving primary care for patients with chronic illness. JAMA.

[7] New York City Department of Health and Mental Hygiene. Take Care New York [Internet].

[8] Kroenke K, Spitzer RL, Williams JB (2001). The PHQ-9: validity of a brief depression severity measure. J Gen Intern Med.

[9] Larson K, Levy J, Rome MG, Matte TD, Silver LD, Frieden TR Public health detailing: a strategy to improve the delivery of clinical preventive services in New York City. Public Health Rep.

[10] (1948). Preamble to the constitution of the World Health Organization as adopted by the International Health Conference [Internet].

